# Complications of colonoscopy surveillance of patients with Lynch syndrome – 33 years of follow up

**DOI:** 10.1007/s10689-024-00416-w

**Published:** 2024-08-05

**Authors:** Alexander Frank, Sophie Walton Bernstedt, Nigin Jamizadeh, Anna Forsberg, Charlotte Hedin, Johannes Blom, Ann-Sofie Backman

**Affiliations:** 1 Dept. of Medicine, Huddinge, Karolinska Institutet, Stockholm, Sweden; 2 Gastroenterology unit, Dept. of Medicine, Capio S:t Görans Hospital, Stockholm, Sweden; 3 Gastroenterology unit, Dept. of Gastroenterology, Dermatovenereology and Rheumatology, Karolinska University Hospital, Stockholm, Sweden; 4 Div. of Upper Gastrointestinal diseases, Karolinska University Hospital, Stockholm, Sweden; 5 Dept. of Clinical Science and Education, Karolinska Institutet, Stockholm, Sweden; 6 Dept. of Clinical Science and Education, Karolinska Institutet, Stockholm, Sweden; 7 Dept. of Surgery, Södersjukhuset, Stockholm, Sweden; 8 Gastroenterology unit, Dept. of Medicine, Ersta Hospital, Stockholm, Sweden

**Keywords:** Lynch syndrome, Colonoscopy, Surveillance, Complications, Safety

## Abstract

Background and study aims: Lynch syndrome (LS) is a hereditary autosomal dominant condition, with an increased lifetime risk of developing malignancies including colorectal cancer (CRC). Current guidelines differ in recommended colonoscopy-surveillance intervals from 1 to 2 years. Although colonoscopy is considered a safe procedure, there are risks of severe adverse events (SAEs), such as perforation and bleeding, as well as adverse events (AEs), such as abdominal discomfort and post-colonoscopy gastrointestinal infections. Colonoscopy-related bleeding and perforation rates have been reported 0.17% and 0.11%, respectively. However, there are insufficient data regarding complications of colonoscopy-surveillance for LS patients. This study aims to investigate the risk of AEs among LS patients during colonoscopy in the Stockholm region. Patients and methods: This retrospective cohort study includes 351 LS patients undergoing endoscopic surveillance at the Karolinska University Hospital, August 1989 – April 2021. Data from endoscopic surveillance colonoscopies were extracted from patients’ medical records. Results: Of 1873 endoscopies in 351 LS patients, 12 complications (AEs) were documented within 30 days (0.64%) and with a total of 3 bleedings (SAEs, 0.16%). No perforations were identified. Conclusion: Colonoscopy surveillance for LS patients shows a comparatively low risk of AEs per-examination. Colonoscopy complications per-patient, including both SAEs and AEs, show a significantly higher risk. Colonoscopy complications only including SAEs, show a comparatively low risk. Understanding the lifetime risk of surveillance-related colonoscopy complications is important when designing targeted surveillance programmes.

## Introduction

Lynch syndrome (LS) is a hereditary autosomal dominant condition, defined and caused by pathogenic germline variants in a DNA mismatch repair (MMR) gene: *MLH1*, *MSH2*, *MSH6*, *PMS2* or deletion of *EpCAM*. Patients with LS have an increased lifetime risk of developing malignancies, e.g., the lifetime risk of colorectal cancer (CRC) and endometrial cancer ranges from approximately 60–80%, compared with 3–4% in the general population [1–3]. Globally, 2–4% of all CRC-cases are considered to be caused by LS [2].

Detection and removal of colorectal adenomas, a potential precursor for developing CRC in LS patients, are consequently important preventive measures in patients with LS [4].

Guidelines differ on the optimal colonoscopy surveillance interval: the American College of Gastroenterology (ACG) recommends a 1-year interval, the European Society for Gastrointestinal Endoscopy (ESGE) recommends a 2-year interval, and the Japanese Society for Cancer of the Colon and Rectum (JSCCR) recommends a 1–2 year interval, starting from the age of 20 to 35 years [5–8].

Colonoscopy is regarded as an important diagnostic and therapeutic tool for diseases of the colon. As CRC-screening and other surveillance programmes (e.g. inflammatory bowel disease, IBD) increase globally, the number of performed colonoscopies and, consequently, the need for colonoscopy increases as well [9]. Colonoscopy is considered a safe procedure [10]. However, when severe adverse events (SAEs) occur (such as bowel perforation, major bleeding or splenic injury), these are associated with higher rates of morbidity and mortality [11].

In a 2016 meta-analysis of complications regardless of colonoscopy cause, the perforation and bleeding rates were estimated at 0.05% and 0.26%, respectively, with a lower rate of complication in screening coloscopies compared with diagnostic or therapeutic colonoscopies [11]. A Swedish population-based register study conducted between 2001 and 2013 of colonoscopies across all indications, reported an incidence rate of 0.17% for major bleeding and 0.11% for bowel perforation [12].

There is evidence that the quality of colonoscopy varies, mainly because of endoscopist- and patient-related factors [13], with less variation in the detection rate of precancerous lesions [14]. Subsequently, increased awareness of the importance of high procedural quality has led to the use of specific quality parameters such as colonoscopy completion rate, adenoma detection rate (ADR), caecal intubation rate (CIR), post-colonoscopy colorectal cancer rate (PCCRC rate), bowel cleanliness defined as Boston Bowel Preparation Scale (BBPS) and rate of colonoscopy-associated complications to define high-quality colonoscopy 15, 16]. Thus, incomplete visualisation of the colonic mucosa, insufficient bowel preparation, and incomplete adenoma removal are factors that increase the risk of PCCRC [14].

LS patients, due to repeated colonoscopies during a lifetime, are potentially at increased risk of developing colonoscopy-related SAEs compared with non-LS patients. In a retrospective study of 300 Japanese LS patients from 2022, the rate of AEs was estimated at 0.2% for colonoscopy surveillance [17]. There are insufficient data regarding safety of colonoscopy surveillance for LS patients in a European setting, and the present study evaluates the safety of colonoscopy surveillance in patients with LS in the Stockholm region, Sweden.

## Materials and methods

### Study design and data collection

This is a multicentre retrospective cohort study of all patients with LS included in a colonoscopy surveillance programme in the Stockholm region, Sweden, from August 1989 to April 2021. In the Stockholm region, Sweden, with a population of 2.4 million people in 2022 [18], there has been an organised surveillance programme for patients with LS since 1989. In total, 5 adjacent colonoscopy centres participated in this study. All emergency hospitals, endoscopic centers and the majority of the healthcare providers in the Stockholm region, Sweden, uses the same regional-based medical record which simplifies the identification of complications. Sweden is known for its renowned quality registries, but currently, a validated registry of LS patients is lacking. National registry data have not been used.

Approximately one-third of the Swedish LS-population are diagnosed at the Karolinska University Hospital, then referred to an endoscopic centre for surveillance colonoscopies. Patients with a registered MMR-mutation according to the InSiGHT Variant Interpretation Committee’s classification [19], were included in the study as previously described [20]. The InSiGHT Variant Interpretation Committee has later been succeeded by the InSiGHT ClinGen Variant Curation Expert Panel [21], however, it was not the predominant classification at the inception of this study.

Data have been retrospectively derived from 1975 to 2021, collected and compiled as a database. Information on age, genotype, sex, smoking history, body mass index (BMI), use of non-steroidal anti-inflammatory drugs (NSAID) and/or aspirin was extracted from patients’ medical records. For a comprehensive descriptive characterisation, the occurrence of certain genotypes has been correlated with the complications. Details of complications of colonoscopy, defined as a complication generating contact of care within 30 days of a performed colonoscopy, were also retrieved from medical records.

### Statistics

Descriptive statistics are presented as mean, median, range, percentage and proportion. IBM SPSS (version 28.0 (190), for Mac) was used for statistical calculations. A p-value of less than 0.05 was considered statistically significant with a confidence interval (CI) of 95%.

### Ethics

This study was approved by the Regional Ethics Review Board of Stockholm, Dnr 2017/2013-31/2 and Dnr 2022-00119-02.

## Results

In total 363 patients who met inclusion criteria (a confirmed MMR-gene deficiency from Karolinska University Hospital or affiliated endoscopic centres, and participating in the colonoscopy surveillance programme) were identified. One patient was excluded from the study because of uncertainty of germline mutation, one patient due to emigration, two patients because of non-implemented planned colonoscopies (never performed any colonoscopies) and eight patients were excluded due to missing data. The remaining patients (*n* = 351) were included in the final analysis with a total of 1873 performed colonoscopies (Table 1). There was no significant difference in the mean age at diagnosis between the different LS-genotypes. The data cover prescription drugs only, and the use of over-the-counter/non-prescription drugs is unknown.

**Table 1:** Patient demographic data, descriptive colonoscopy data

Study population, *n*	351	
**Colonoscopies**,** n**	1873	
**Sex**,** n (%)**	Female	190 (54)
	Male	161
**Genotype**,** n (%)**	*MLH1*	158 (45)
	*MSH2*	98 (28)
	*MSH6*	49 (14)
	*PMS2*	37 (10,5)
	*EpCAM*	2 (0,5)
	*Mixed genotype*	7 (2)
**Age at diagnosis (mean ± SD)**	*MLH1*	41,6 ± 15
	*MSH2*	40 ± 13
	*MSH6*	42,9 ± 16
	*PMS2*	47,5 ± 17
	*EPCAM*	50,5 ± 9
	*Mixed genotype*	44 ± 12
**Colonoscopy intervals**,** days**	Median	487
	IQR	386–687
**Colonoscopies per patient**	Median	4
	IQR	1–6
**Antiplatelet therapy**	ASA (Trombyl)	18*
	ASA (Treo)	3
	ASA (unknown dose)	3
	NSAID	10
	Cox-2 inhibitor	1
*75 mg (*n* = 16), 150 mg (*n* = 1), 160 mg (*n* = 1)	All	35

### Complications

**Fig. 1:**
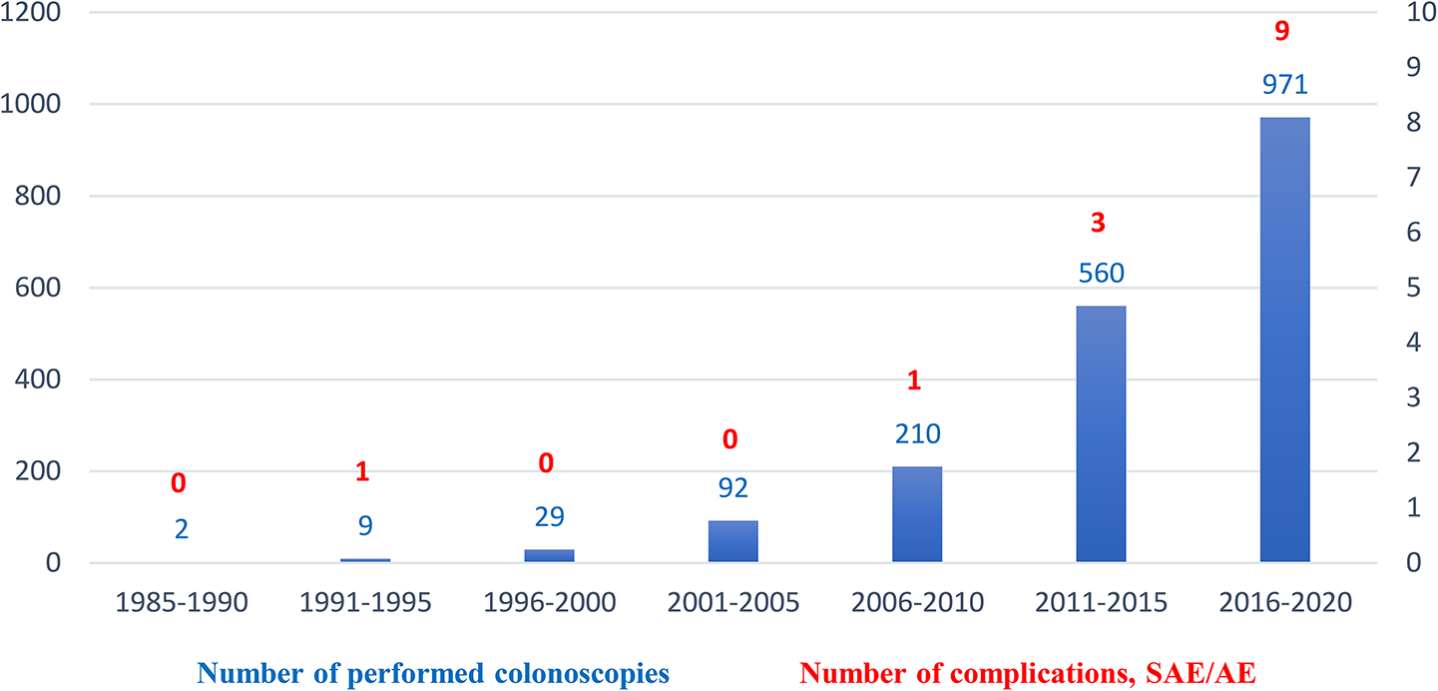
Patients with verified Lynch Syndrome included in colonoscopy surveillance in the Stockholm region, Sweden: number of performed colonoscopies and complications

A total of 12 complications were observed, with 3 severe AEs (SAE) (25.0%; bleeding, *n* = 3) and 9 AEs (75.0%; abdominal pain, *n* = 7; gastrointestinal (GI) infections, *n* = 2) (Table 2). One perforation in a LS patient was identified but occurred 4 years before a verified LS-diagnosis, and inclusion in the organised surveillance programme, and was therefore excluded due to study criteria. In addition, at the time of this perforation (1991), the diagnosis Lynch Syndrome was interchangeably used with the initial term Hereditary Nonpolyposis Colorectal Cancer (HNPCC), according to a criteria-based system which may contribute to a classification error. One patient with abdominal pain was excluded, as the AE occurred 4 years before start of the surveillance programme. Age at complication for SAEs was 67 years (median, range 55.2–78.8), AEs 58.9 (range 45.3–72.5; abdominal pain 57.7, range 44.8–70.6); GI-infections 62 (range 41.5–82.5) and for all complications 62.4 (range 49.5–75.3). There was a median 7.8 years’ interval (range 3.5–12.1) from diagnosis to all complications: 9 years for SAEs (range 8–10); 7.3 years for AEs (range 2.4–12.2); abdominal pain 7.9 (range 2.3–13.5); GI-infections 5.5 (range 4.8–6.2). There were 3 patients on antiplatelet therapy (acetylsalicylic acid, 75 mg), of which 2 experienced SAEs (bleeding) and 1 AE (GI-infection). Five patients had undergone a therapeutic colonoscopy (polypectomy; bleeding, *n* = 2; GI-infection, *n* = 2; abdominal pain, *n* = 1) and 3 of 5 (60%) patients had antiplatelet therapy drugs (acetylsalicylic acid 75 mg). The complications occurred within 6 days (mean, range 0–15) after performed colonoscopy. Of the reported complications, *MSH2* was the most frequent MMR-mutation (*MSH2*, *n* = 7; *MLH1*, *n* = 4; *MSH6*, *n* = 1). Of all complications, 6 of 12 (50%) occurred in the year 2020 with a majority (92%, *n* = 11) after 2010 (Fig. 1).

**Table 2:** Characteristics of complications

Complications, *n*	12	
**SAEs/Severe adverse events**,** n**	Bleeding	3*
	Perforation	0
**Type of examination**,** n (%)**	Therapeutic	2^†^ (67)
	Diagnostic	1 (33)
**AEs/Adverse events**,** n (%)**	Abdominal pain	7 (78)
	GI-infections	2 (22)
**Type of examination**,** n (%)**	Therapeutic	3^¥^ (33)
	Diagnostic	6 (66)

* Clopidogrel (*n* = 1), ASA 75 mg (*n* = 1)

^†^ polypectomy (*n* = 2)

^¥^ polypectomy (*n* = 2), ESD (*n* = 1)

The risk of bleeding per-examination was 0.16%. Risk of SAE, based on risk per-examination regardless of genotype was 0.16%. Risk of AE and SAE, based on risk per-examination regardless of genotype, was 0,64%.

Risk of SAE, based on risk per-patient regardless of genotype, was 0.85%. Risk of AE and SAE, based on risk per-patient regardless of genotype, was 3.41%.

## Discussion

The aim of this study was to explore the safety of colonoscopy surveillance for LS patients. The results of our study, for both SAEs and AEs, suggest an overall low complication rate, which are consistent with anticipates outcomes and comparable to those reported in similar studies.

Within the scope of this paper: AEs have been categorised as abdominal pain/discomfort or infectiously caused gastrointestinal symptoms e.g. loose stools or diarrhea. SAEs have been categorised as bleeding or perforation. The risk of bleeding for an LS patient *per-examination* in a surveillance programme, in comparison with a non-LS patient, is lower with a relative risk reduction of 0.06%, compared with results from Forsberg et *al*. (0.17%). There was no perforation (0%) in this study in comparison with Forsberg et *al*. (0.11%).

The rate of both AEs and SAEs for *each* LS patient are significantly higher. Over a life-time, the cumulative risk of complications for each LS patient is likely to be higher than for populations not enrolled in colonoscopy surveillance programmes.

As demonstrated, the majority of the complications occurred after 2011. Most presumably, this reflects better colonoscopy registrations for subsequent years rather than inferiorly performed colonoscopies. However, it cannot be neglected, that the colonoscopy technique has been improved during the timeframe of this study. For instance, the use of carbon dioxide instead of air for colonoscopy insufflation, an increasingly dominant method since the turn of the millennium, has demonstrated its advantages in regards to e.g. abdominal discomfort [22, 23]. It is noteworthy, as seen in Fig. 1, that the complications registered from 2016 to 2020 corresponds to approximately 1/100 in regards to colonoscopy-associated complications per-examination. From a Swedish context, there are unfortunately deficiencies in reporting, such as desaturation during examination. To provide a relatively fair picture, the decision was therefore made to exclude cardiovascular complications.

There are currently classifications to facilitate the characterisation of complications. The Classification for Adverse Events Gastrointestinal Endoscopy (AGREE) classification, provides a novel adapted tool specifically designed for endoscopy [24], although not widely used in clinical practice. As a result of still limited use, the decision was made not to use the AGREE classification.

Colonoscopy surveillance for LS patients aims to detect and remove potential precancerous adenomas, based on the assumption that surveillance-programmes are patient-safe, feasible and have a significant impact on CRC-mortality. In other words, the potential benefits of colonoscopy surveillance for LS patients must outweigh the risks. Due to the low average number of lifetime colonoscopies, the cumulative complication rate experienced by the overall population is low.

LS patients on the other hand, as a cohort, requiring recurrent annual or biennial colonoscopies, have an increased cumulative risk of colonoscopy-associated complications. Since LS patients are younger, healthier, and, hence, less prone to other comorbidities, the colonoscopy-associated complication rates become increasingly important. AEs and perforations, in particular, are of specific interest as they are affiliated with higher mortality- and morbidity rates [11].

For LS patients, colonoscopy-associated complications have long-term consequences beyond the immediate mortality and morbidity. These complications include reduced trust in the provided care, fear of future colonoscopies, and a reduced propensity to follow surveillance programmes with consequent risk of reduced adherence. Of note, LS patients’ adherence to surveillance programmes has been reported to be inadequate, partly due to patients’ perceived challenges associated with colonoscopy, including discomfort, embarrassment and the time-consuming nature of the procedure [25–27]. Patients experience bowel preparation as burdensome [28], particularly as LS patients must undergo bowel preparation repeatedly [29]. The first colonoscopy experience may determine the patient’s adherence to the surveillance programme [23]. An increased frequency of hospitalisation and follow-ups also increase health economic impacts.

It is well-known that the risk of the development of CRC differs between LS-genotypes. Other factors affecting the risk of CRC include sex, BMI, smoking history and non-participation in regular colonoscopy surveillance that contributes to the risk of CRC [19, 30, 31].

Historically, former guidelines on colonoscopy surveillance for LS patients in Sweden and Europe did not consider the LS-genotype when designing surveillance programmes [32]. This implies, that CRC-risk is unevenly distributed, but the colonoscopy-related complications are not, which is troublesome from a benefit–risk perspective [33]. Consequently, the colonoscopy surveillance programme should be targeted and individualised, and based on certain preconditions. However, on the basis of surveillance programmes’ arrangement, only a fraction of the surveillance programme’s participants will benefit. Moreover, this possible presumed benefit may vary for each participant into only modest longevity.

To our best knowledge, this is the first study of its kind, providing an assessment of colonoscopy surveillance-associated complications and colonoscopy safety in a Swedish setting of a large cohort consisting of 351 LS patients over 33 years of age. LS, as previously described, is a rare condition which presents challenges in the gathering of larger LS cohorts. Although LS patients represent a small subgroup of those undergoing colonoscopy, repeated colonoscopies increase individual LS patients’ lifetime risk, highlighting the importance of these data. Furthermore, the overall high-quality population data in Sweden regarding colonoscopy complications partially compensates for the lack of a direct comparison group of the study.

There are several limitations to this study. First, it is unknown to what extent the number of referred LS patients to Karolinska University Hospital corresponds with the actual number of LS patients in the Stockholm region. Hence, it is possible that there are colonoscopies as well as colonoscopy-associated complications not captured in the study. Moreover, there may be differences between the patients referred and not referred. Second, data were acquired from only one region in Sweden with mainly White individuals, which may decrease generalisability. Third, like any other retrospective cohort study, there may be limited- or missing data and, accordingly, there is risk of less accurate entry of data which may affect overall data quality.

Future studies should address whether individualised screening programmes affect the relative and absolute risk of surveillance-related colonoscopy complication for LS patients. In addition, studies comparing complication rates between centres with a high volume compared with a low volume of LS patient surveillance colonoscopies, would determine whether centralisation of surveillance programmes would impact complication rates.

In conclusion, this study demonstrates that per-examination colonoscopy complication rates in LS patients are comparable to those previously reported in the general population. However, understanding the lifetime risk of surveillance-related colonoscopy complications is important when designing targeted surveillance programmes, as LS patients require lifelong surveillance. The use of more specific and targeted programmes could increase cost-effectiveness and further positively affect the risk–benefit balance.
